# Integrin β3 organizes dendritic complexity of cerebral cortical pyramidal neurons along a tangential gradient

**DOI:** 10.1186/s13041-020-00707-0

**Published:** 2020-12-14

**Authors:** Brian D. Swinehart, Katherine M. Bland, Z. Logan Holley, Andrew J. Lopuch, Zachary O. Casey, Christopher J. Handwerk, George S. Vidal

**Affiliations:** Department of Biology, James Madison University, 951 Carrier Drive, Harrisonburg, VA 22801 USA

**Keywords:** Integrin beta 3, Integrin β3, Itgb3, Dendritic complexity, Dendrite, Pyramidal neuron, Dendritogenesis, Autism, In utero electroporation, Mosaic analysis

## Abstract

Dysfunctional dendritic arborization is a key feature of many developmental neurological disorders. Across various human brain regions, basal dendritic complexity is known to increase along a caudal-to-rostral gradient. We recently discovered that basal dendritic complexity of layer II/III cortical pyramidal neurons in the mouse increases along a caudomedial-to-rostrolateral gradient spanning multiple regions, but at the time, no molecules were known to regulate that exquisite pattern. Integrin subunits have been implicated in dendritic development, and the subunit with the strongest associations with autism spectrum disorder and intellectual disability is integrin β3 (*Itgb3*). In mice, global knockout of *Itgb3* leads to autistic-like neuroanatomy and behavior. Here, we tested the hypothesis that *Itgb3* is required for increasing dendritic complexity along the recently discovered tangential gradient among layer II/III cortical pyramidal neurons. We targeted a subset of layer II/III cortical pyramidal neurons for *Itgb3* loss-of-function via Cre-loxP-mediated excision of *Itgb3*. We tracked the rostrocaudal and mediolateral position of the targeted neurons and reconstructed their dendritic arbors. In contrast to controls, the basal dendritic complexity of *Itgb3* mutant neurons was not related to their cortical position. Basal dendritic complexity of mutant and control neurons differed because of overall changes in branch number across multiple branch orders (primary, secondary, etc.), rather than any changes in the average length at those branch orders. Furthermore, dendritic spine density was related to cortical position in control but not mutant neurons. Thus, the autism susceptibility gene *Itgb3* is required for establishing a tangential pattern of basal dendritic complexity among layer II/III cortical pyramidal neurons, suggesting an early role for this molecule in the developing brain.

## Introduction

Typically, neuroanatomical studies of neurological disorders have limited their analyses to specific brain regions. For example, while excitatory pyramidal dendritic development is clearly impaired among a wide variety of neuropsychiatric disorders—including Rett syndrome [2], *MeCP2*: [64], Angelman syndrome (*Ube3a*: [51], autism (*TAOK2*: [6]; *Epac2*: [73]), and others (BTBR model: [8]; *CYFIP1*: [61]—it is not known whether the deficits are confined to the areas studied or if a much larger deficit is present (for example, over a large area of the cerebral cortex).

We recently demonstrated that dendritic complexity is arranged along a gradient in layer II/III excitatory pyramidal neurons of the mouse cortex [31]. This finding in the mouse is similar to observations in primate species, including humans, where multiple adjacent, functionally-related regions (e.g., V1, V2, V4, TEO) show a clear caudal-to-rostral increase in dendritic complexity, a change that is thought to alter their functional properties (reviewed by [20–22, 32]. Similarly, dendritic spine density and number generally increases in a caudal-to-rostral direction across multiple regions [18, 19].

We wanted to test whether these gradients of dendritic complexity and spine density are regulated by autism risk genes. We chose integrin β3 (*Itgb3*) for our study because a substantial body of literature associates *Itgb3* as a quantitative trait locus for autism spectrum disorder and intellectual disability [12, 13, 46, 56, 58, 69, 72, 80]. Global knockout of *Itgb3* in mice leads to autistic-like behaviors [7] and neuroanatomical deficits [17]. However, *Itgb3* is also critical for platelet function, so knockout mice often hemorrhage during fetal development and adulthood, leading to low viability [7, 30]. Other studies have pointed to an important role for *Itgb3* in midbrain [15, 50, 59, 75, 81], hippocampus [10, 37, 63], and in cortical neurons in vitro [33]. Furthermore, various integrin subunits have been implicated in dendritic outgrowth [34, 41, 79] and *Itgb3* specifically may be involved in *Thy1*-mediated neurite retraction in vitro [29], but, to our knowledge, a putative role for *Itgb3* in dendritic arborization has not been explored in vivo.

To answer the question of whether or not Itgb3 plays a role in dendritic complexity and spine density, we specifically targeted layer II/III excitatory pyramidal neurons across large swaths of the mouse cerebral cortex for deletion of *Itgb3 *in vivo, utilizing approaches and control data established in our prior work [31]. Our results point to a cell-specific requirement for *Itgb3*, an autism risk gene, in establishing a normal gradient of dendritic complexity and spine density in excitatory pyramidal neurons across the developing mouse cerebral cortex.

## Materials and methods

### Mice and in utero electroporation

**Fig. 1:**
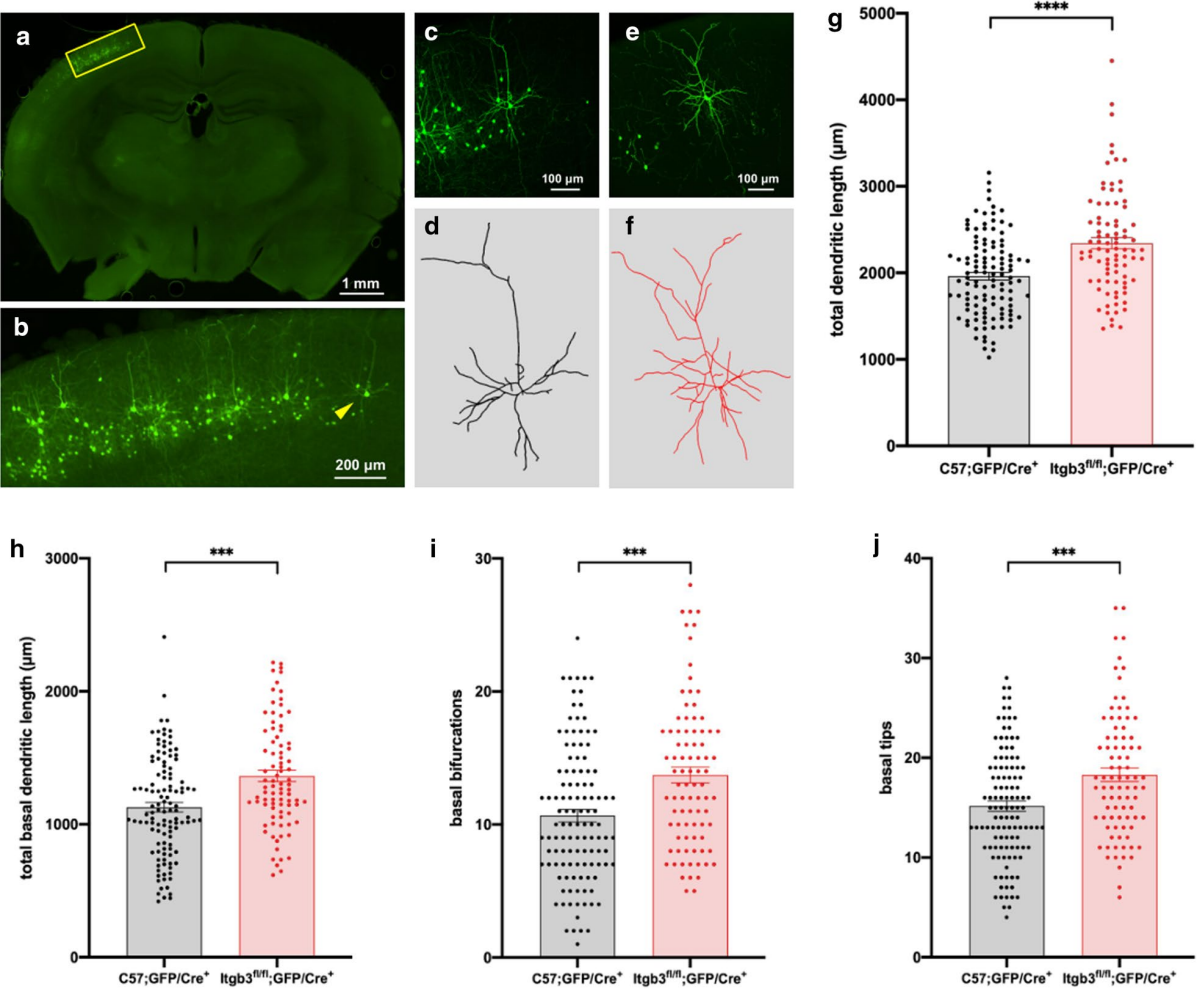
*Itgb3*^*fl/fl*^;GFP/Cre^+^ layer II/III cortical pyramidal neurons exhibit higher dendritic complexity than C57;GFP/Cre^+^ neurons. **a** Example of a low-magnification (× 4) image taken of a coronal section from a C57 mouse with labeled GFP/Cre^+^ neurons. **b** Magnified view of the inset in (**a**), showing the targeting specificity of GFP-labeled, layer II/III excitatory pyramidal neurons. **c** Medium-magnification (× 20) maximum intensity Z-projection from confocal images of the neuron identified in (**b**). **d** A 3D reconstruction of all apical and basal dendrites of the neuron in (**c**). **e**, **f** As in (**c**, **d**), this time of an *Itgb3*^*fl/fl*^;GFP/Cre^+^ neuron. **g** Total dendritic length is higher in *Itgb3*^*fl/fl*^;GFP/Cre^+^ neurons (2342 ± 64.50 μm) when compared to C57;GFP/Cre^+^ neurons (1962 ± 42.90 μm). **h** Total basal dendritic length is higher in *Itgb3*^*fl/fl*^;GFP/Cre^+^ neurons (1363 ± 42.71 μm) when compared to C57;GFP/Cre^+^ neurons (1128 ± 34.98 μm). **i** Number of basal bifurcations (branches) is higher in *Itgb3*^*fl/fl*^;GFP/Cre^+^ neurons (13.72 ± 0.59) when compared to C57;GFP/Cre^+^ neurons (10.67 ± 0.48). **j** Number of basal tips (terminal ends of basal dendrites) is higher in *Itgb3*^*fl/fl*^;GFP/Cre^+^ neurons (18.29 ± 0.68) when compared to C57;GFP/Cre^+^ neurons (15.16 ± 0.52). C57;GFP/Cre^+^ neurons were previously reported in Holley et al. [31] and re-analyzed for this study. C57;GFP/Cre^+^ N = 116 neurons; *Itgb3*^*fl/fl*^;GFP/Cre^+^ N = 86 neurons. ****p < 0.0001, ***p < 0.001, Mann–Whitney

To determine whether *Itgb3* expression in excitatory pyramidal neurons is required for normal dendritic morphology across multiple cortical regions, we studied C57BL/6-*Itgb3*^*tm1.1Wlbcr*^/J mice (“*Itgb3*^*fl/fl*^”, Jackson Labs #028232, [55] with C57BL6/J mice (“C57”, Jackson Labs #000664) as controls. (In Additional File 1: Figure 1, we additionally studied *Itgb3*^*fl/f*l^ mice crossed with B6.129S2-*Emx1*^*tm1(cre)*Krj^/J mice, Jackson Labs #005628 [27]). Husbandry and housing was done as previously described [31]. C57 and *Itgb3*^*fl/fl*^ mice were maintained as separate lines. We targeted GFP/Cre constructs to newborn developing layer II/III excitatory pyramidal neurons via in utero electroporation at embryonic day 15.5, as described previously [4, 31]. In the *Itgb3*^*fl/fl*^ line, exon 1 of *Itgb3* is flanked by loxP sites, and Cre recombinase expression causes rapid and robust excision [23, 55].

To ensure strong GFP and Cre recombinase expression in targeted neurons, we used the “Supernova” system [45, 54] as described previously [4, 31]. To summarize, in the “Supernova” approach, two constructs are co-electroporated in utero. One construct contains a tetracycline response element (TRE)-Cre recombinase sequence, electroporated at a final concentration of 10 μg/mL, and the other consists of a CAG-loxP-stop-loxP-green fluorescent protein (GFP)-tetracycline trans-activator (tTA) sequence, electroporated at a final concentration of 1 mg/mL. We electroporated 1.0 μL of this solution into one lateral ventricle of each experimental animal and targeted developing layer II/III pyramidal neurons by using a large (5 mm) electrode placed over the dorsal telencephalon at embryonic day 15.5 [31]. Whenever TRE-Cre is present within a neuron, it is very likely that multiple copies of CAG-loxP-stop-loxP-GFP-tTA are present, as it was co-electroporated at a 100-fold higher concentration than TRE-Cre. In this case, leaky Cre expression from the TRE-Cre construct causes excision of the STOP site from the other construct, leading to the expression of both GFP and tTA. These constructs then act in a positive feedback manner leading to high levels of expression of GFP, Cre recombinase, and tTA [45, 54]. Conversely, excision in the CAG-loxP-stop-loxP-GFP-tTA construct without TRE-Cre does not occur spontaneously in vivo [45, 54], meaning that GFP labeling requires the presence of both constructs.

Apart from the above strategy—designed for robust Cre recombinase and GFP expression in targeted neurons—other experimental advantages of in utero electroporation have been described previously [4, 31]. In sum, they are: (1) limited selection bias compared to other methods, as nearly all labeled neurons can be analyzed, (2) targeting of neuronal precursors fated to become layer II/III neurons [25], increasing the cell type specificity of the experiment, (3) targeting of S- and M-phase neural progenitors within a 6–8 h window [31, 74], (4) targeting a large swath of the developing telencephalon, allowing dozens of neurons to be studied within the same brain [31], and (5) targeting a sparse population of neurons, creating a sparse mosaic where mutant neurons develop in a “sea of wild-type neurons and glia” [76].

### Overall experimental controls

This study was carried out in accordance with established ethical principles and approved institutional protocols (see Declarations, below). Subjective bias and reproducibility were controlled as in Holley et al. [31]. In brief, we minimized selection bias by acquiring images blind to morphological characteristics and exact anatomical position, and coding sample names. The experiment included a total of 202 pyramidal neurons (C57;GFP/Cre+ N = 116 neurons, *Itgb3*^*fl/fl*^;GFP/Cre+ N = 86 neurons). Six independent and complete replications of the experiment (i.e., brains derived from separate in utero electroporation surgeries, and thus from separate litters) were used (C57;GFP/Cre+ brain 1 N = 24 neurons, brain 2 N = 45 neurons, brain 3 N = 47 neurons; *Itgb3*^*fl/fl*^;GFP/Cre+ brain 1 N = 13 neurons, brain 2 N = 30 neurons, brain 3 N = 43 neurons). C57;GFP/Cre+ neuron reconstructions used as controls in this study were taken from the NeuroMorpho repository and were previously published [31], though C57,GFP/Cre+ and *Itgb3*^*fl/fl*^;GFP/Cre+ neurons were both initially collected together as part of the same experiment. Males and females were both used, and data from each genotype were pooled. Brains were selected for their broad rostrocaudal pattern of GFP expression. All reconstructed neurons will be housed as .swc files in the NeuroMorpho repository, http://NeuroMorpho.org ([1, 3] see Availability of data and materials, below).

### Histology, microscopy, and analysis

Histological, microscopy, and analysis procedures were performed as previously described [31]. In brief, to capture dendritic morphology of labeled neurons after dendritic arborization is mostly complete [35, 48, 52, 62], we perfused and fixed brains at postnatal day 23, sectioned them coronally (100 µm thick). We then mounted the sections on slides with Prolong Diamond Antifade Mountant (Life Technologies), cured them, and sealed them. We identified GFP-positive layer II/III cortical pyramidal neurons and imaged their dendritic arbors with a Nikon Eclipse TE2000-E confocal microscope (20 × /0.75 NA), at or above the Nyquist sampling rate. We used low-power (4 × /0.20 NA) images to determine the distance of each neuron from the midline and from the cortical surface. We identified the cortical region of each neuron by registering anatomical landmarks from the low-power images to the 2008 Allen Mouse Brain Reference Atlas [38]. In the case of regional analysis (Additional file 1: Figure 4), we only included neurons with a high confidence of being within a cortical region (i.e., > 0.25 mm from the closest neighboring region), and a sufficient sample size for comparison was found in primary somatosensory cortex. We created semi-automated 3D reconstructions from the 20 × images using neuTube software [24] and the Simple Neurite Tracer plugin for FIJI [44], creating .swc files. We analyzed the reconstructions with L-Measure software [70]. Some reconstructions were recalibrated manually to ensure that branch orders would be counted correctly by L-measure.

### Principal axis calculation and statistics

We modeled the tangential cortical position of each neuron along a “principal axis” [5], as in Holley et al. [31], where caudomedial neurons are closer to the principal axis origin, and rostrolateral neurons are further away. To summarize, we assigned Cartesian coordinates to each neuron in microns, comprising a mediolateral position *x*_1_ (i.e., the distance to midline, where *x*_0_ = 0 μm) and rostrocaudal position y_1_. (In Additional file 1: Figure 3, a geodesic (or “encephalodesic”) mediolateral distance *x*_1_ was calculated by tracing along the pial surface of the cortex from the midline to a point radially superficial to the neuron). We calculated a “distance to principal axis origin” *z* as follows: *z* = √((*x*_*1*_)^2^ + (*m*(*y*_*1*_ − *y*_*0*_))^2^), where *m* is an arbitrary, unitless scalar and *y*_*0*_ is an arbitrary rostrocaudal position (in μm). The total dendritic length of each neuron was plotted against *z*, and we iteratively modified m and y_0_ until the maximum goodness of fit to a linear model (r^2^) was achieved. In the case of C57;GFP/Cre+ neurons, these values were *m* = 1.56, *y*_0_ = − 350 μm [31]. Once these parameters were established, other anatomical features besides total dendritic length (e.g., basal dendritic length) were plotted against *z*. We performed all statistical analyses and graphing with GraphPad Prism software.

### Dendritic spine density microscopy and analysis

Basal dendritic branches of GFP-positive layer II/III cortical pyramidal neurons whose dendritic arbors were previously imaged at 20 × for 3D reconstruction and analysis were re-imaged on a Nikon Eclipse TE2000-E confocal microscope at 60 ×/1.40NA. We took Z-stack confocal images for analysis of dendritic spine type and density. Pixel size (i.e., sampling) within the confocal software (Nyquist XY and 1–4 × Nyquist Z) determined the size of the image to optimize resolution. Beginning with the first basal dendrite clockwise of the apical dendrite, we imaged approximately 170 μm of each basal dendrite (totaling an average of 710 μm dendritic length imaged per cell). We analyzed images blind to condition to determine dendritic lengths and dendritic spine count/type using a semi-automated image analysis software (NeuronStudio), as this approach confers an advantage in consistency and reliability of results [65, 67]. Dendritic spines were classified into three categories: stubby, thin, or mushroom [28]. Dendritic spine density was calculated by dividing the total number of spines (or, in the case of spine type, the total number of stubby, thin, or mushroom spines) imaged from each cell by the total dendritic length imaged from each cell.

## Results

**Fig. 2:**
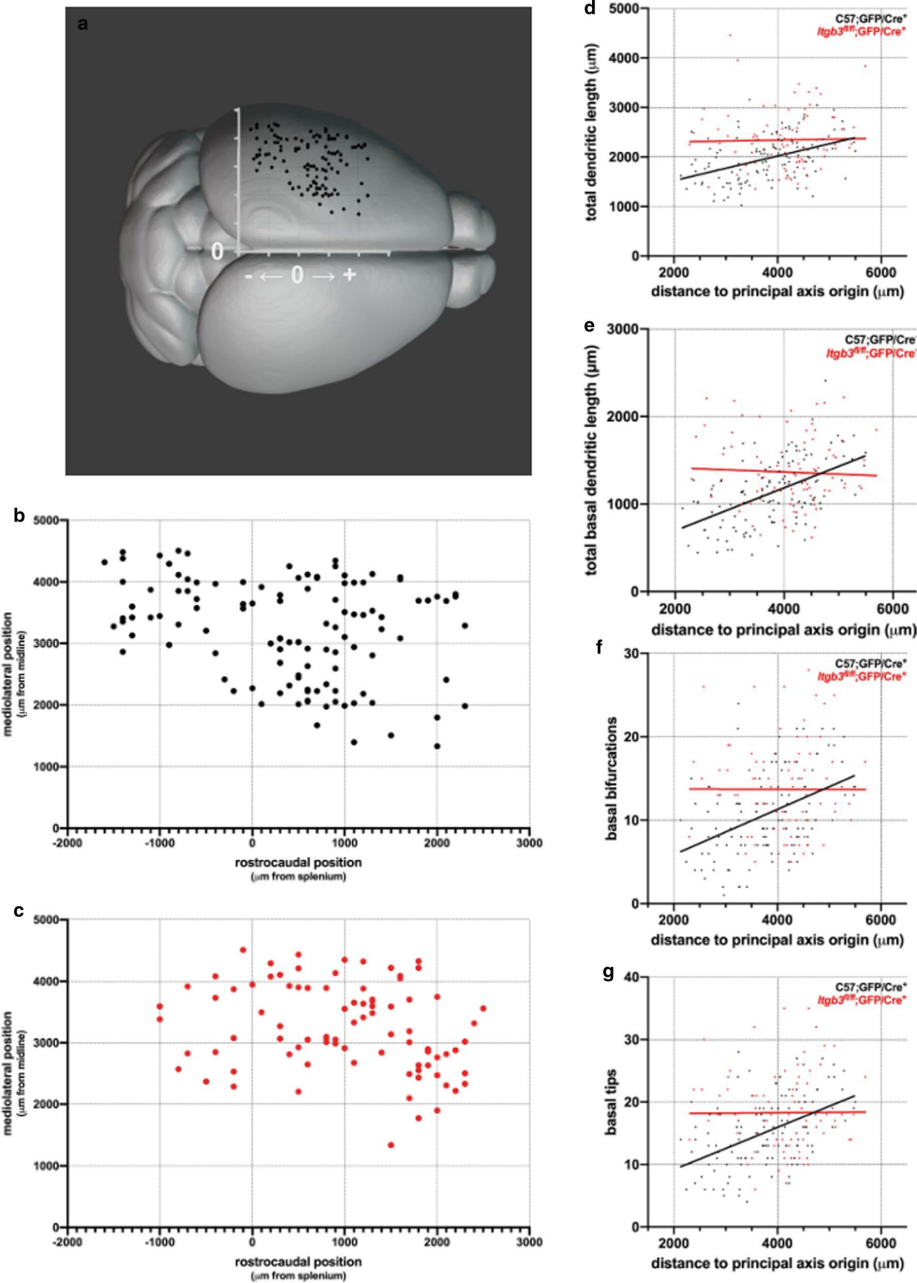
In contrast to *Itgb3*^*fl/fl*^;GFP/Cre^+^ neurons, tangential cortical positions of C57;GFP/Cre^+^ layer II/III neurons are correlated with dendritic complexity. **a** 3D schematic showing cortical (x,y) positions of neurons in (**b**) over a dorsal view of the mouse brain. The x-axis measures distance away from the splenium of the corpus callosum (positive numbers are rostral), and the y-axis measures distance away from the midline. **b** Cortical (x,y) positions of C57;GFP/Cre^+^ neurons. **c** Cortical (x,y) positions of *Itgb3*^*fl/fl*^;GFP/Cre^+^ neurons. **d** Total dendritic length is significantly correlated to cortical position along a tangential gradient among layer II/III C57;GFP/Cre^+^ neurons, but not among *Itgb3*^*fl/fl*^;GFP/Cre^+^ neurons (linear regression; respective regression slopes are significantly different). **e** Total basal dendritic length, **f** number of basal bifurcations, and **g** number of basal tips are all correlated to cortical position along a tangential gradient among C57;GFP/Cre^+^ but not *Itgb3*^*fl/fl*^;GFP/Cre^+^ neurons. In each graph, the linear regression slopes between C57;GFP/Cre^+^ and *Itgb3*^*fl/fl*^;GFP/Cre^+^ are significantly different. R^2^ and p-values for (**g**–**j**) are summarized in Table 1. C57;GFP/Cre^+^ neurons were previously reported in Holley et al. [31] and re-analyzed for this study. 3D model of mouse brain © 2017 Allen Institute for Brain Science, Allen Mouse Common Coordinate Framework (CCFv3) [78]. Available from: atlas.brain-map.org. C57;GFP/Cre^+^ N = 116 neurons; *Itgb3*^*fl/fl*^;GFP/Cre^+^ N = 86 neurons

We generated isolated *Itgb3* mutant layer II/III neurons that developed among wildtype neurons and glia, permitting us to study the cell-specific effect of *Itgb3* on dendritic complexity in vivo, when compared to controls. To target newborn layer II/III neurons, we combined in utero electroporation with Cre-loxP recombination [4]. As expected, in utero electroporation in both C57 and *Itgb3*^*fl/fl*^ mice targeted somata that migrated to layer II/III of cortex (Fig. 1a). Electroporated cells display a pyramidal neuronal morphology (Fig. 1b–f), and these neurons were distributed across a large extent of the tangential plane of the cerebral cortex (Fig. 2). Cre-targeted neurons in the cortex of Itgb3^fl/fl^ show a decrease in integrin β3 expression by P23 (Additional file 1: Figure 1). The Itgb3^fl/fl^ line is on the C57 genetic background, and no differences in dendritic complexity are seen among layer II/III pyramidal neurons between unmanipulated C57 and Itgb3^fl/fl^ mice at P23 (Additional file 1: Figure 1). All dendritic arbors of each neuron analyzed for this study, regardless of genotype, were extensively labeled with GFP (Fig. 1c, e), permitting accurate morphological reconstruction and analysis.

We observed that the mean total dendritic length of mutant neurons is 19% greater than controls (p < 0.0001, Fig. 1g). Mutants had 17% higher apical dendritic length than controls (p = 0.02, not shown) as well as 21% higher basal dendritic length (p = 0.0001, Fig. 1h). Dendritic length could arise from longer segments or more segments per cell. We found that both branching (“bifurcations”) and terminal ends (“tips”) were higher in mutants, as well (p < 0.001, Fig. 1i, j).

### Cortical position predicts dendritic complexity among control but not *Itgb3* mutant layer II/III pyramidal neurons

Neurons were distributed across large areas of the cerebral cortex, so we measured their mediolateral and rostrocaudal positions (Fig. 2a–c) to test whether the cortical position of a neuron was predictive of its dendritic complexity. The (*x*,*y*) position of each neuron is its distance to the midline along the mediolateral (*x*) axis and its rostrocaudal (*y*) distance relative to the splenium, with positive numbers being rostral to the splenium and negative numbers being caudal to the splenium (Fig. 2a). In prior work [31], we were observed a correlation between cortical position and dendritic complexity among control C57;GFP/Cre^+^ neurons by calculating a single value for each neuron: its distance to a principal axis origin (0 μm, − 350 μm).

Using this paradigm, we observed another major difference between *Itgb3* mutant and control neurons: The dendritic length of control (C57;GFP/Cre^+^) but not mutant (*Itgb3*^*fl/fl*^;GFP/Cre^+^) layer II/III pyramidal neurons is correlated to their respective cortical positions (Fig. 2d, Table 1). In controls, caudomedial layer II/III pyramidal neurons generally have a shorter total dendritic length, and moving rostrolaterally, total dendritic length increases (Fig. 2d; [31]). But in mutants, caudomedial layer II/III pyramidal neurons are comparatively longer than caudomedial controls, and advancing rostrolaterally, total dendritic length does not increase (Fig. 2d, Table 1). This result is also found when comparing correlations between cortical position and basal dendritic length, basal bifurcations, or basal tips (Fig. 2e, f, and g, respectively; Table 1).

**Table 1:** R^2^, p-values, and N for Figs. 2 and 3

	C57; GFP/Cre^+^ R^2^	C57; GFP/Cre^+^ p-value	*Itgb3*^*fl/fl*^; GFP/Cre^+^ R^2^	*Itgb3*^*fl/fl*^; GFP/Cre^+^ p-value	Do re-gression slopes differ? p-value
Figure 2d	0.185	< 0.0001	< 0.001	.9746	0.0087
Figure 2e	0.271	< 0.0001	0.004	0.5579	< 0.0001
Figure 2f	0.181	< 0.0001	< 0.001	0.995	0.0038
Figure 2g	0.234	< 0.0001	< 0.001	0.876	0.0022
Figure 3a	0.168	< 0.0001	0.015	0.270	0.1229
Figure 3b	0.028	0.701	0.053	0.033	0.5511
Figure 3c	0.235	< 0.0001	< 0.001	0.9588	0.0012
Figure 3d	0.032	0.056	0.025	0.143	0.0182
Figure 3e	0.148	< 0.0001	0.002	0.7045	0.0157
Figure 3f	0.057 (N = 113)	0.011	< 0.001	0.919	0.0957
Figure 3g	0.126	< 0.0001	0.003	0.6292	0.0044
Figure 3h	0.079 (N = 95)	0.006	< 0.001 (N = 84)	0.558	0.0248

**Fig. 3:**
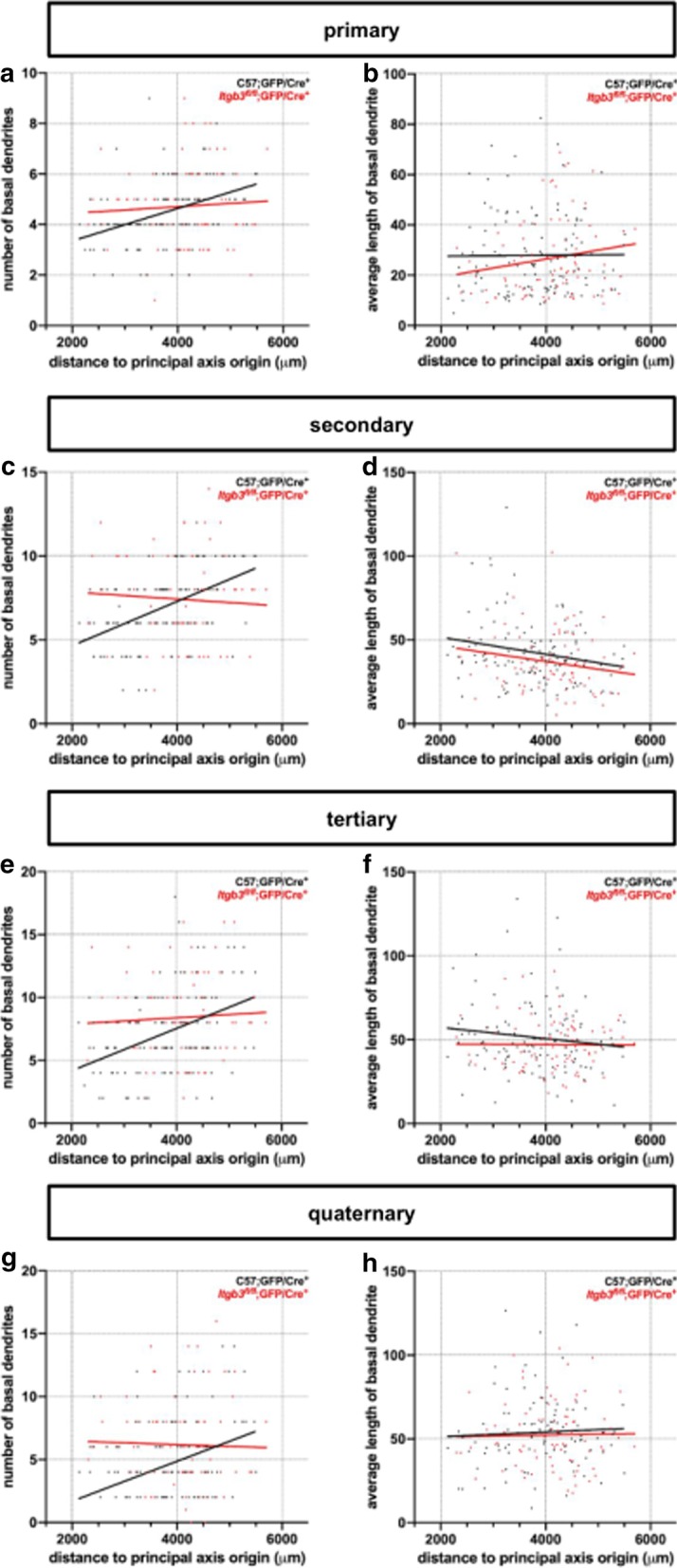
Changes in dendritic complexity come from changes in basal dendrite number (but not length) at multiple branch orders. **a** The number of primary basal dendrites is correlated to cortical position along a tangential gradient among C57;GFP/Cre^+^ neurons but not *Itgb3*^*fl/fl*^;GFP/Cre^+^ neurons. **b** No correlation between average length of primary basal dendrites and cortical position along a tangential gradient among either C57;GFP/Cre^+^ or *Itgb3*^*fl/fl*^;GFP/Cre^+^ neurons. **c**, **d** As in (**a**, **b**), in secondary basal dendrites. **e**, **f** As in (**a**, **b**), in tertiary basal dendrites. **g**, **h** As in (**a**, **b**), in quaternary basal dendrites. R^2^ and p-values for (**a**–**h**) are summarized in Table 1. C57;GFP/Cre^+^ neurons were previously reported in Holley et al. [31] and re-analyzed for this study. C57;GFP/Cre^+^ N = 116 neurons; *Itgb3*^*fl/fl*^;GFP/Cre^+^ N = 86 neurons, unless otherwise noted in Table 1

**Fig. 4:**
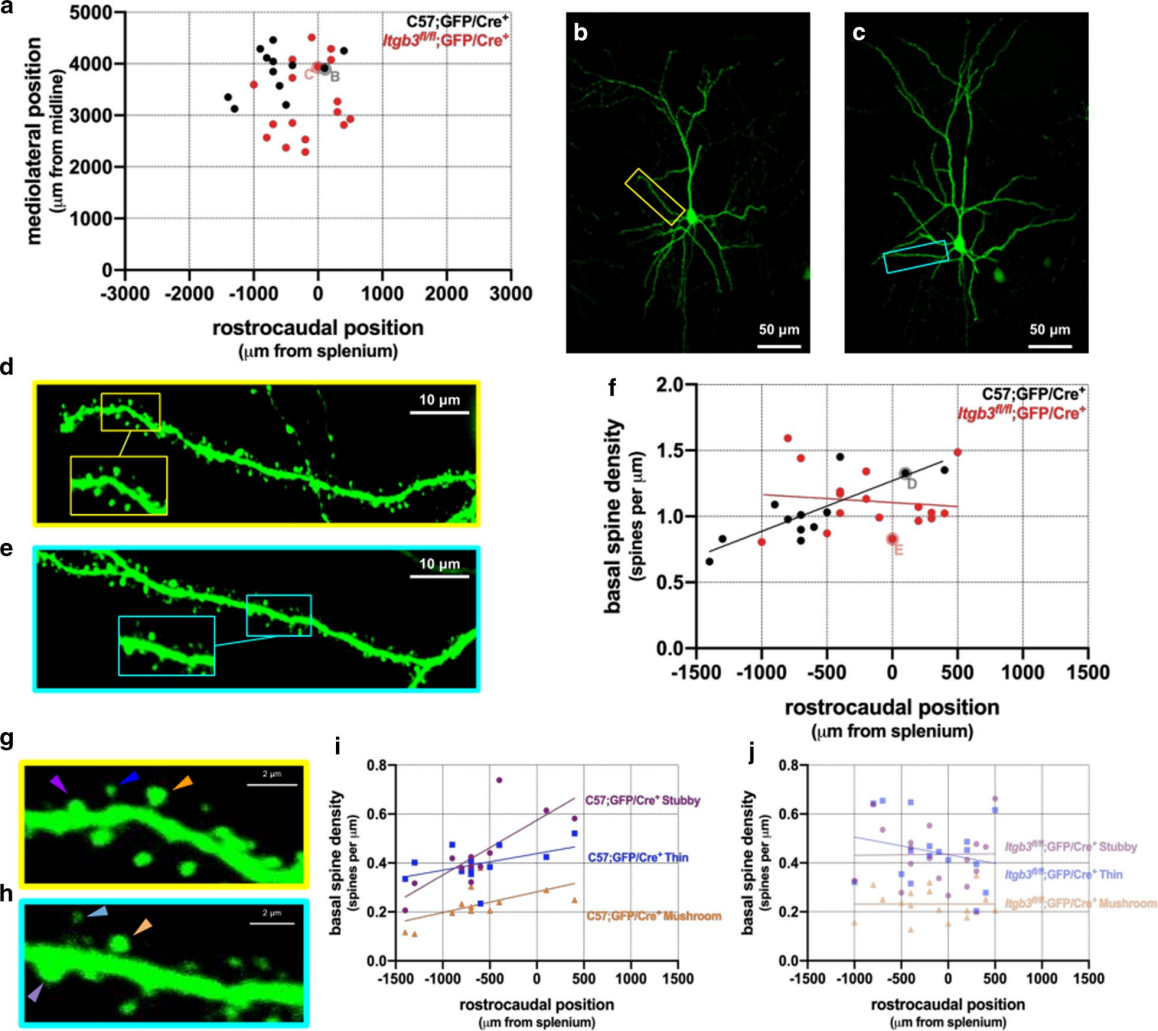
In contrast to *Itgb3*^*fl/fl*^;GFP/Cre^+^ neurons, rostrocaudal positions of C57;GFP/Cre^+^ layer II/III neurons are correlated with basal dendritic spine density. **a** Cortical (x,y) positions of C57;GFP/Cre^+^ neurons (black) and *Itgb3*^*fl/fl*^;GFP/Cre^+^ neurons (red). **b** Medium magnification (× 20) maximum intensity Z-projection of C57;GFP/Cre^+^ neuron (black dot labeled “B” in panel **a**). **c** As in (**b**), this time of a *Itgb3*^*fl/fl*^;GFP/Cre^+^ neuron (red dot labeled “C” in panel **a**). **d** High magnification (× 60) maximum intensity Z-projection of a basal dendrite (yellow inset in panel **b**). **e** As in (**d**), of cyan inset in panel **c**. **f** In C57;GFP/Cre^+^ neurons, basal dendritic spine density is strongly correlated with rostrocaudal position (R^2^ = 0.74, p < 0.006), but not in *Itgb3*^*fl/fl*^;GFP/Cre^+^ neurons (R^2^ = 0.013, p = 0.66). **g** Magnified view of inset in panel **d**, showing the three main dendritic spine types: mushroom (red arrowhead), thin (blue arrowhead), and stubby (orange arrowhead). **h** As in (**g**), of inset in panel **e**: mushroom (light red arrowhead), thin (light blue arrowhead), and stubby (light orange arrowhead). **i** Basal dendritic spine type density by rostrocaudal position in C57;GFP/Cre^+^ neurons. R^2^ = 0.65, p < 0.003 (stubby), R^2^ = 0.45, p = 0.13 (thin), R^2^ = 0.76, p = 0.05 (mushroom); **j** Rostrocaudal position of *Itgb3*^*fl/fl*^;GFP/Cre^+^ neurons is not correlated with dendritic spine density of any spine type. R^2^ < 0.001, p = 0.95 (stubby), R^2^ < 0.06, p = 0.36 (thin), R^2^ < 0.00001, p = 0.99 (mushroom). C57;GFP/Cre^+^ N = 12 neurons; *Itgb3*^*fl/fl*^;GFP/Cre^+^ N = 17 neurons

Several possibilities exist for the apparent lack of relationship between tangential cortical position and dendritic complexity among *Itgb3* mutant neurons. For example, dendritic complexity could be related to a *different* gradient of tangential cortical position among mutant neurons, or there may be no relationship at all. To distinguish between these possibilities, we modified the tangential gradient model for *Itgb3* mutant neurons (“principal axis”, see 'Materials and methods'; [5, 31]), which includes both mediolateral and rostrocaudal data. As in controls [31], mediolateral or rostrocaudal position alone were not predictive of dendritic morphology among *Itgb3* mutant neurons. But, to our surprise, no combination of rostrocaudal and mediolateral positioning data in the principal axis revealed any important (e.g., R^2^ > 0.05) or significant (e.g., p < 0.05) relationship between cortical position and dendritic complexity among mutants (Additional file 1: Figure 2). The mouse cerebral cortex is curved, so it is possible that dendritic complexity could also depend on the position of a neuron along the dorsoventral axis in addition to the mediolateral and rostrocaudal axis. To infer dorsoventral positioning from coronal sections, we re-measured the mediolateral distance of each neuron by tracing along the pial surface of the cortex from the midline to the point radially superficial to its cell body. This results in geodesic (or, more properly, “encephalodesic”) measurements of distance to midline. Taking these measurements into account slightly modifies the correlation between neuronal position and dendritic complexity, but again fails to reveal any important or significant relationship among *Itgb3* mutant neurons (Additional file 1: Figure 3). Control and *Itgb3* mutant neurons were located in multiple cortical regions, and the primary somatosensory cortex contained a sufficient sample size in both groups to test how dendritic complexity varies within a cortical region. As demonstrated previously, cortical region is not related to dendritic complexity in the mouse brain [31]. Here, we also observed that no overall differences exist between control and *Itgb3* mutant neurons within the same cortical region (Additional file 1: Figure 4). Another possibility is that somatic depth or cortical region play a role in the relationship between cortical position and dendritic complexity among *Itgb3* mutant neurons. However, both *Itgb3* mutant and control somata reach normal depth ranges for layer II/III (Additional file 1: Figure 5A), and the in utero electroporation approach already targets neurons with a very specific laminar fate (see 'Materials and methods'). At the same time, the range of somatic depths found in this layer in both control and mutant neurons raises the possibility that even slight overall changes in depth could bias the study. To address this concern, we restricted the dataset to the middle of layer II/III by including only somata located 250–300 μm from the cortical surface. As with the full dataset (Fig. 2d), there is a significant relationship between tangential cortical position and dendritic complexity among control but not *Itgb3* mutant neurons (Additional file 1: Figure 5B). To account for cortical region, we restricted our dataset to neurons residing within the primary somatosensory cortex. This region was chosen because a sufficient sample size was available to compare correlations among both control and mutant *Itgb3* neurons. The relationships between cortical position and dendritic complexity found in the full dataset (Fig. 2d) were maintained within the primary somatosensory region (Additional file 1: Figure 5C).

### Correlations between cortical position and dendritic complexity differ between *Itgb3* mutant and control neurons because of changes in basal dendritic complexity

The changes we observed in total dendritic length (Fig. 1g) could come from changes in apical dendritic length, basal dendritic length, or a combination of both. We observed that basal dendritic length is better related to cortical position than total dendritic length among controls (Fig. 2d, e, Table 1). In fact, basal dendritic length nearly doubles in controls when comparing the most rostrolateral neurons to the most caudomedial neurons in the sample (Fig. 2d, e). Among *Itgb3* mutant neurons, however, there is no relationship between basal dendritic length and cortical position (Fig. 2e, Table 1). Basal dendritic branching (measured as either the total number of bifurcations or the total number of terminal ends) is also related to cortical position in control but not *Itgb3* mutant neurons (Fig. 2f, g). On the other hand, apical dendritic length and apical dendritic branching are not related to cortical position in either *Itgb3* mutant or control neurons (Additional file 1: Table 1).

### Differences in basal dendritic length between *Itgb3* mutants and controls come from changes in dendritic branching, not average segment length

The alterations in basal dendritic length (Fig. 2d, e) and branching (Fig. 2f, g) that we observed could arise in several ways. For example, the total basal dendritic length and branch number of a neuron could increase 20% by simply adding 20% more primary basal dendrites to the neuron, as long as each basal dendrite had similar parameters (such as average segment length and higher-order branching; [53]). We therefore analyzed the number of branches and the average segment length at each branch order (primary, secondary, etc.), starting with the segments closest to the soma (primary dendrites) and moving centrifugally.

Overall, primary, secondary, tertiary, and quaternary basal dendritic branching is correlated to the somatic cortical position in control but not *Itgb3* mutant neurons. Basal dendritic branching among caudomedial control neurons is lower than among mutants, but moving rostrolaterally, basal dendritic branching increases in control neurons at all four branch orders (primary: Fig. 3a, secondary: Fig. 3c, tertiary: Fig. 3e, and quaternary: Fig. 3g; Table 1). This relationship was not apparent among any higher-order (quinary, senary, and septenary) dendrites (Additional file 1: Table 1). Also, the average segment length at all branch orders did not generally vary with cortical position in either condition (Fig. 3b, d, f, h, Table 1).

Taken together, it is clear that the tangential gradient of dendritic complexity among controls is driven by variations in branching rather than in average segment length. It is also clear that this phenomenon is completely abrogated among *Itgb3* mutant neurons.

### Rostrocaudal position is correlated with basal dendritic spine density in control but not *Itgb3* mutant layer II/III pyramidal neurons

Elevated dendritic complexity in a neuron could increase its total dendritic spine number if dendritic spine density remains the same. GFP labeling was sufficient not only for accurately reconstructing dendritic arbors, but also for counting dendritic spine density, particularly along basal dendrites (Fig. 4b–e). We were able to test whether cortical position is correlated with basal dendritic spine density in control and *Itgb3* mutant neurons. To do so, we sampled from neurons in a specific (*x*,*y*) area (Fig. 4a) and imaged approximately 170 μm of each of their basal dendrites (Fig. 4d, e), resulting in an average of 710 μm dendritic length imaged per cell.

We found that the basal dendritic spine density of control neurons were correlated with their rostrocaudal position (Fig. 4f), but that *Itgb3* mutant neurons were not (Fig. 4f). Among control neurons, we found that mediolateral position is not important in predicting dendritic spine density, which is in contrast to dendritic complexity, where both rostrocaudal and mediolateral position are determining factors. The spine density changes we observed in controls could be due to changes in one or more dendritic spine types (e.g., stubby, thin, and/or mushroom). To test this, we categorized our data by spine type (Fig. 4g, h) and found that all three dendritic spine type densities tended to increase rostrocaudally in control neurons (Fig. 4i), though this was not a significant correlation in some spine types (stubby p < 0.003, thin p = 0.13, mushroom p = 0.05). On the other hand, spine type densities on *Itgb3* mutant neurons displayed no apparent correlation to rostrocaudal position (Fig. 4j).

## Discussion

Our findings strongly suggest a cell-specific role for *Itgb3* in controlling the dendritic complexity and spine density of developing cortical excitatory pyramidal neurons in vivo. *Itgb3* mutant layer II/III pyramidal neurons in mosaic cerebral cortical tissue appear to be “insensitive” to a tangential gradient of arborization. In other words, *Itgb3* is essential for establishing a gradient of dendritic complexity among layer II/III pyramidal neurons of the cerebral cortex.

### Cell-autonomous control of dendritic complexity and spine density along a tangential gradient?

This study revealed *Itgb3* function by producing a mosaic in which neurons with *Itgb3* loss-of-function developed in vivo surrounded by wildtype tissue. This sparse mosaic (targeting on the order of 0.01% of all neurons: see [31]) shows that *Itgb3* is needed to regulate dendritic complexity in a cell-specific manner. We are hesitant, however, to conclude that this novel function of *Itgb3* is cell-autonomous. A fully cell-autonomous function would be abrogated in neurons with *Itgb3* loss-of-function (which we have demonstrated) but would be intact among untargeted synaptic partners or neighbors (which we have not tested). However, it is difficult to conceive a simple mechanism for how *Itgb3* loss-of-function within a neuron would influence its own basal dendritic complexity while also exerting a notable non-cell-autonomous function on its neighbors’ dendritic arbors. In other words, while we cannot definitively conclude that *Itgb3* function in dendritic arborization is fully cell-autonomous, it is a very likely explanation of the data.

Itgb3 appears to control dendritic complexity along a tangential gradient that involves mediolateral and rostrocaudal positioning, but *Itgb3* appears to control dendritic spine density along a rostrocaudal gradient. Regardless of whether *Itgb3* controls dendritic complexity and spine density in a cell-autonomous or cell-specific manner, how can it set separate tangential gradients across the cortex for two apparently separate morphological phenomena? In other words, if *Itgb3* regulates both dendritic complexity and spine density, their distinct gradients could suggest that *Itgb3* may have two distinct functions in developing cortical pyramidal neurons. However, little is known about the underlying mechanisms; one possibility is differential expression and activation of integrin β3 across the cortex during development. Integrin β3 forms a functional heterodimer exclusively with αv in the brain [60] and several ligands of integrin αvβ3, including RGD-peptide containing ligands like fibronectin and vitronectin, are expressed in the brain [39]. Furthermore, it is known that RGD peptides activate neuronal integrin β3, increasing dendritic spine density in hippocampus over a 2 week period, while specifically blocking integrin β3 also blocks the spine density increase [71]. Perhaps integrin β3 and/or its ligands are expressed in a graded fashion throughout the cortex. For example, a rostrocaudal change in RGD-containing extracellular matrix proteins (e.g., fibronectin, vitronectin) may activate integrin β3 in differing degrees. Spine density changes due to integrin β3 activation have been shown to occur only after 2 weeks of activation in vitro [71], whereas other studies manipulating integrin β3 in vitro for shorter periods of time do not show any changes in spine density (2 days: [9], 5 days: [33]). Unfortunately, the tangential and laminar (let alone developmental) patterns of expression of integrin β3 and RGD-containing extracellular matrix proteins in vivo are not currently known. Nonetheless, it is known that other extracellular matrix proteins (e.g., aggrecan, brevican) are indeed differentially expressed [11, 14]. Understanding the expression of RGD-containing extracellular matrix proteins in the brain and how they activate integrin β3 in vivo may be an important path to uncover the mechanisms for how *Itgb3* regulates dendritic complexity and spine density.

### A specific, compartmentalized function for *Itgb3* during dendritogenesis

Changes to dendritic complexity across the mouse cortex are driven by *Itgb3*-mediated changes in branching, rather than changes in average segment length. However, a major way to control the dendritic field size of a neuron is by regulating mean dendritic segment length. In both humans and rodents, mean dendritic segment length generally increases by branch order among cortical pyramidal neurons [40, 53, 66], hippocampal granule neurons [42, 43, 77], and apical and basal dendrites of hippocampal pyramidal neurons [77]. This pattern is visible in basal dendrites of controls (Fig. 2b, d, f, h). Interestingly, *Itgb3* does not appear to regulate this pattern, so it is likely that dendritic segment length and dendritic branching are independently regulated.

Our data show that the number of primary dendritic branches per neuron increases across the cortex in a caudomedial to rostrolateral direction (Fig. 2a) and is regulated by *Itgb3*. This increase is proportionally similar in secondary, tertiary, and quaternary dendrites (Fig. 2c, e, g). In theory, increasing the number of primary basal dendrites on a neuron would also increase the higher order branches by a similar proportion. Thus, our findings therefore imply (though they do not demonstrate) that it is the *primary basal dendritic number* that may be the major regulatory function of *Itgb3* during dendritogenesis. Primary dendrites are fully established by the end of the 1st week of life, before secondary branching occurs [35, 48, 62, 82]. *Itgb3* may be involved in dendritic overgrowth and/or pruning mechanisms in layer II/III pyramidal neurons. For example, dendritic overgrowth and pruning has been demonstrated in vivo in adult-born hippocampal granule neurons [26]. Analogously, it is plausible that similar mechanisms shape the later morphological development of cortical pyramidal neurons, although there is no evidence that specific branch orders are specifically targeted, as they appear to be in our data. Thus, *Itgb3* may have an early, rather than late, function in dendritogenesis.

An open question is whether *Itgb3* controls dendritic arborization through a direct mechanism (e.g., by activating cytoskeletal regulators during dendritic arborization), or if we observed a compensatory effect. Because these events necessarily occur earlier than the developmental timepoint we studied (postnatal day 23; P23), we cannot conclude definitively about the exact time in which *Itgb3* is required for normal dendritic development. This is also because there is currently no reliable method to measure the expression of integrin β3 in electroporated neurons during development, although we were able to detect an analogous decrease in integrin β3 expression by P23 in cortex using a conditional *Itgb3* KO (Additional file 1: Figure 1). On the other hand, we did focus our experiment early, at P23, soon after dendritic arborization is normally complete [35, 48, 52, 62], suggesting that a compensatory mechanism would have very little time to influence arborization patterns, regardless of the actual timing of integrin β3 deletion in *Itgb3* mutant neurons. Thus, we posit that *Itgb3* control of dendritic arborization may be a primary, and not compensatory, effect.

### Consequences of *Itgb3* dysfunction in the cerebral cortex

The loss of a tangential gradient of dendritic complexity and spine density has clear implications for brain function in specific cortical regions. Within the human somatosensory cortex, dendritic complexity in laterally-positioned neurons, devoted to hand and finger sensation, have more branched dendrites when compared to medial neurons devoted to trunk sensation [68]. In this study, we did not know the precise sensory function of each targeted neuron, so the consequences of losing a tangential gradient of dendritic complexity within functional regions remain to be tested. For example, if the normal caudomedial-to-rostrolateral variation in dendritic complexity within the primary somatosensory cortex (Additional file 1: Figure 1C) were mapped to exact somatosensory functions, then predictions could be made about how *Itgb3* loss of function would affect somatosensory processing.

Dendritic outgrowth in pyramidal neurons is an anatomical substrate for excitatory tone. In the neocortex, dendritic structure of pyramidal neurons is intimately linked with their excitability [47]. The altered pattern of dendritic arborization of layer II/III pyramidal neurons with *Itgb3* loss-of-function may modify their connectivity in ways that alter the overall tangential pattern of excitatory-inhibitory balance in the cortex (as proposed by Nelson and Valakh [57]). A tangential pattern of excitatory-inhibitory balance has been observed across hierarchically-organized regions of the visual cortex [16]. Furthermore, in many models of neurodevelopmental disorders, altered excitation and altered anatomical characteristics of cortical pyramidal neurons go hand-in-hand. For example, DSCAM model mice have impaired neocortical pyramidal dendritic arborization [49] as well as reduced neocortical excitability in vivo [36]. Thus, losing an important overall anatomical pattern among *Itgb3* mutant neurons may also have concomitant physiological and behavioral aberrations, as well.

## Conclusion

We observed that *Itgb3* is essential for establishing gradients of dendritic complexity and spine density in layer II/III pyramidal neurons of the mouse cortex. We pinpointed the change in dendritic complexity to a change in dendritic branching rather than dendritic length, at various branch orders (primary, secondary, etc.). Furthermore, because only labeled neurons were targeted for *Itgb3* excision (i.e., targeted neurons developed in a “sea” of wildtype neurons), we conclude that these functions of *Itgb3* are cell-specific. Overall, our observations support the idea that *Itgb3*, an autism risk gene, has an essential, cell-specific function in establishing an anatomical gradient among excitatory neurons of the living cerebral cortex.

Of course, *Itgb3* is not the only known autism risk gene. It is possible, and perhaps even likely, that many other risk genes for neurodevelopmental disorders also regulate the exquisite microanatomical organization of dendritic arborization and excitatory circuitry across the tangential plane of the cerebral cortex. Our findings, therefore, provide an innovative avenue to advance the fundamental understanding of the molecules regulating excitatory cerebral cortical development.

## Supplementary information

**Additional file 1: Figure 1.** (A-C) No difference in number of primary basal dendrites between C57;GFP/Cre- and *Itgb3*^*fl/fl*^;GFP/Cre- neurons. **Figure 2.** Maximum correlation coefficient (*r*^*2*^) values of total dendritic length versus distance *z* to principal axis origin, as a function of *m* or *y*_*0*_. **Figure 3.** Incorporating geodesic (“encephalodesic”) distance to midline slightly modifies correlations of neuronal position and dendritic complexity in C57;GFP/Cre^+^ and *Itgb3*^*fl/fl*^;GFP/Cre^+^ layer II/III pyramidal neurons. **Figure 4.** Dendritic complexity of C57;GFP/Cre^+^ and *Itgb3*^*fl/fl*^;GFP/Cre^+^ layer II/III pyramidal neurons in the primary somatosensory cortex. **Figure 5.** Somatic depth and region are not important factors when comparing dendritic morphology among targeted layer II/III neurons. **Figure 6.** In contrast to rostrocaudal position (Fig. 4), no correlation exists between total basal dendritic spine density and distance to principal axis origin (*m* = 1.56, *y*_*0*_ = − 350 μm) in either C57;GFP/Cre^+^ neurons (*r*^*2*^ = 0.11, p = 0.30) or *Itgb3*^*fl/fl*^;GFP/Cre^+^ neurons (r*2* = 0.18, p = 0.09). **Table 1.** Apical morphology and high-order basal dendritic morphology of neurons are not correlated to their cortical position along a tangential gradient (distance to principal axis origin).

## Data Availability

The datasets generated and analyzed will be available as .swc files in the NeuroMorpho repository, http://NeuroMorpho.org [1, 3]. The reconstruction data taken from C57,GFP/Cre+ neurons analyzed and re-analyzed for this study are from a previously published study [31] and are also freely available on NeuroMorpho.
