# Supplementary material for: Integrin β3 organizes dendritic complexity of cerebral cortical pyramidal neurons along a tangential gradient
Source: Mol Brain. 2020 Dec 14;13:168. doi: 10.1186/s13041-020-00707-0 (PMC7734815; doi:10.1186/s13041-020-00707-0)
Supplement: Supplementary file 1 — Additional file 1: Figure 1. (A-C) No difference in number of primary basal dendrites between C57;GFP/Cre- and Itgb3fl/fl;GFP/Cre- neurons. Figure 2. Maximum correlation coefficient (r2) values of total dendritic length versus distance z to principal axis origin, as a function of m or y0. Figure 3. Incorporating geodesic (“encephalodesic”) distance to midline slightly modifies correlations of neuronal position and dendritic complexity in C57;GFP/Cre+ and Itgb3fl/fl;GFP/Cre+ layer II/III pyramidal neurons. Figure 4. Dendritic complexity of C57;GFP/Cre+ and Itgb3fl/fl;GFP/Cre+ layer II/III pyramidal neurons in the primary somatosensory cortex. Figure 5. Somatic depth and region are not important factors when comparing dendritic morphology among targeted layer II/III neurons. Figure 6. In contrast to rostrocaudal position (Fig. 4), no correlation exists between total basal dendritic spine density and distance to principal axis origin (m = 1.56, y0 = − 350 μm) in either C57;GFP/Cre+ neurons (r2 = 0.11, p = 0.30) or Itgb3fl/fl;GFP/Cre+ neurons (r2 = 0.18, p = 0.09). Table 1. Apical morphology and high-order basal dendritic morphology of neurons are not correlated to their cortical position along a tangential gradient (distance to principal axis origin). [file 13041_2020_707_MOESM1_ESM.docx]

## Additional Figures, Table, and Figure Legends

### Additional Figure 1


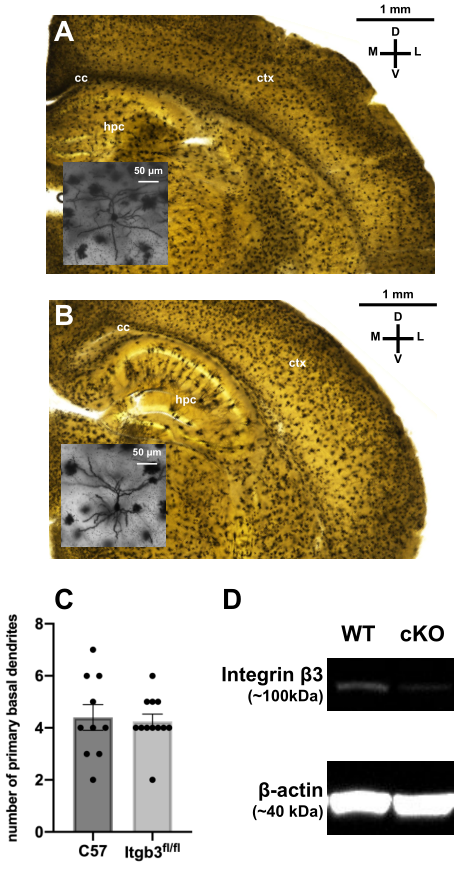


**Additional Figure 1. (A-C) No difference in number of primary basal dendrites between C57;GFP/Cre- and *Itgb3^fl/fl^*;GFP/Cre- neurons. (A)** Example of a low-magnification (4×) image taken of a coronal section from a C57 mouse, stained with the Golgi-Cox method. Inset: medium-magnification (20×) image of a labeled layer II/III pyramidal neuron. **(B)** As in (A), this time from a *Itgb3^fl/fl^* mouse. **(C)** In the visual cortex, there is no difference in the number of primary basal dendrites between C57 and *Itgb3^fl/fl^* neurons. D, dorsal; V, ventral; M, medial; L, lateral; cc, corpus callosum; ctx, cortex; hpc, hippocampus. N = 10 (C57), 12 (Itgb3^fl/fl^) neurons. **(D)** **Itgb3 protein expression is lower in cerebral cortex of conditional knockout mice (cKO: *Emx1*-Cre;*Itgb3^fl/fl^*) when compared to WT mice (C57).** Each lane was loaded with 46 µg of protein extracted from the cerebral cortex of WT or cKO, respectively.

### Additional Figure 2


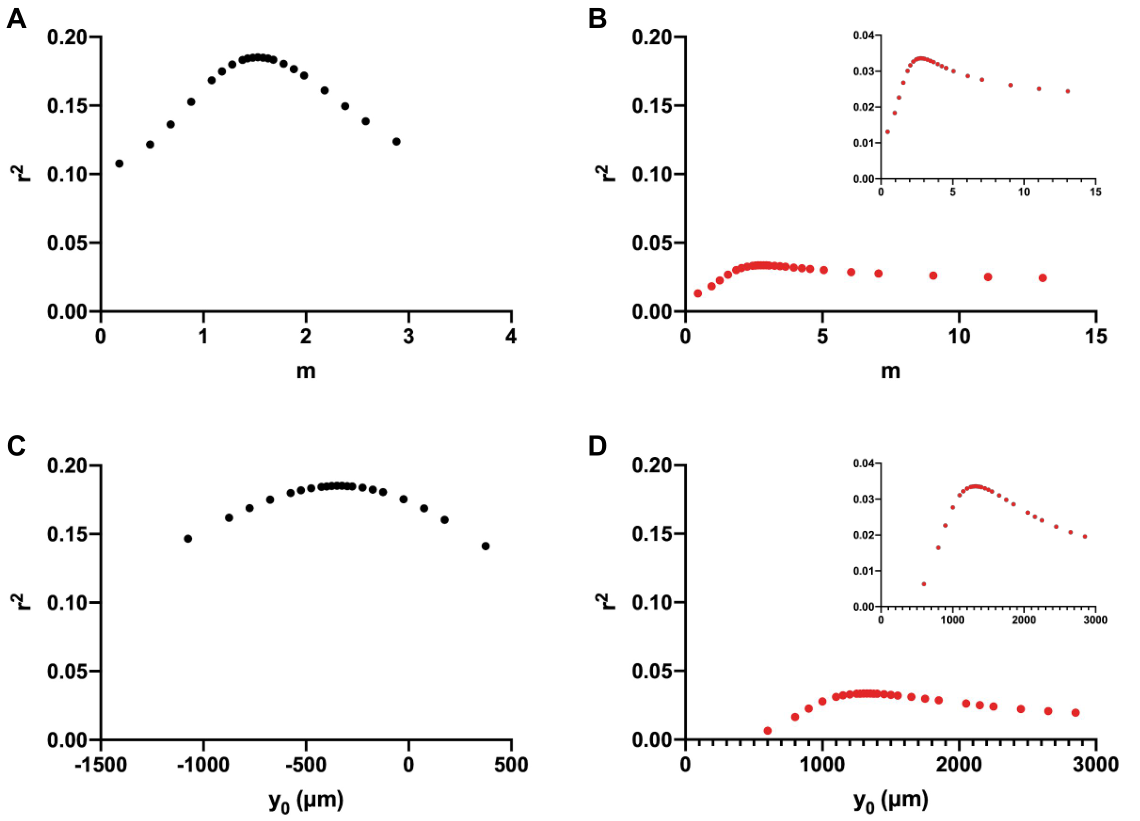


**Additional Figure 2. Maximum correlation coefficient (*r^2^*) values of total dendritic length versus distance *z* to principal axis origin, as a function of *m* or *y_0_*. (A)** *r^2^* *versus m* from C57;GFP/Cre^+^ data (*y_0_* constant at −350 μm). **(B)** As in (A), this time with *Itgb3^fl/fl^*;GFP/Cre^+^ data. Inset: y-axis range reduced to show maximum. **(C)** *r^2^* *versus m* from C57;GFP/Cre^+^ data (*m* constant at 1.56). **(D)** As in (C), this time with *Itgb3^fl/fl^*;GFP/Cre^+^ data. Inset: y-axis range reduced to show maximum.

### Additional Figure 3


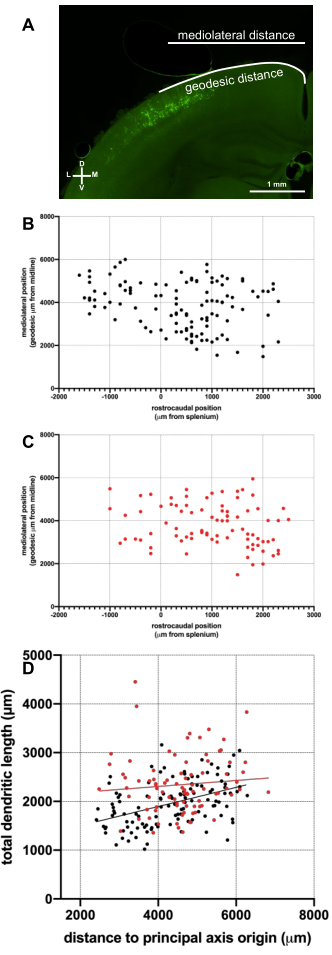


**Additional Figure 3. Incorporating geodesic (“encephalodesic”) distance to midline slightly modifies correlations of neuronal position and dendritic complexity in C57;GFP/Cre^+^ and *Itgb3^fl/fl^*;GFP/Cre^+^ layer II/III pyramidal neurons. (A)** Coronal section from Figure 1A demonstrating geodesic versus mediolateral distance measurements. **(B)** Cortical (*x,y*) positions of C57;GFP/Cre^+^ neurons, where y uses geodesic rather than mediolateral distance measurements. **(C)** As in (B), cortical (*x,y*) positions of *Itgb3^fl/fl^*;GFP/Cre^+^ neurons. **(D)** Total dendritic length is significantly correlated to cortical position along a tangential gradient among layer II/III C57;GFP/Cre^+^ neurons, but not among *Itgb3^fl/fl^*;GFP/Cre^+^ neurons (C57;GFP/Cre^+^ *r^2^* = 0.195, p < 0.0001; *Itgb3^fl/fl^*;GFP/Cre^+^ *r^2^* = 0.008, p = 0.40). C57;GFP/Cre^+^ neurons were previously reported in Holley et al. (2018) and re-analyzed for this study. C57;GFP/Cre^+^ N = 116 neurons; *Itgb3^fl/fl^*;GFP/Cre^+^ N = 86 neurons. D, dorsal; V, ventral; M, medial; L, lateral.

###
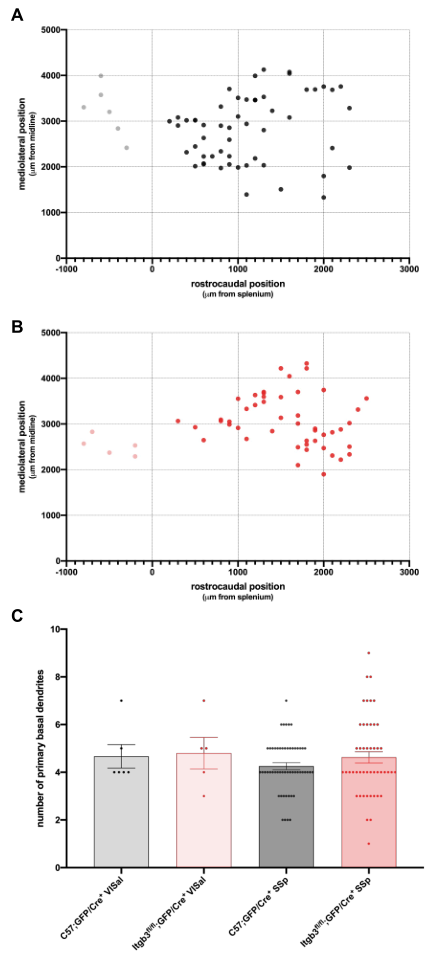
Additional Figure 4

**Additional Figure 4. Dendritic complexity of C57;GFP/Cre^+^ and *Itgb3^fl/fl^*;GFP/Cre^+^ layer II/III pyramidal neurons in the primary somatosensory cortex. (A)** Cortical (*x,y*) positions of C57;GFP/Cre^+^ neurons in anterolateral visual cortex (VISal, gray) and primary somatosensory cortex (SSp, black). **(B)** Cortical (*x,y*) positions of *Itgb3^fl/fl^*;GFP/Cre^+^ neurons in VISal (pink) and SSp (red). **(C)** Dendritic complexity is not different across regions or conditions (Kruskal-Wallis, p = 0.55). C57;GFP/Cre^+^ neurons were previously reported in Holley et al. (2018) and re-analyzed for this study. N = 6 (C57;GFP/Cre^+^ VISal), 5 (*Itgb3^fl/fl^*;GFP/Cre^+^ VISal), 55 (C57;GFP/Cre^+^ SSp), 48 (*Itgb3^fl/fl^*;GFP/Cre^+^ SSp) neurons.

### Additional Figure 5


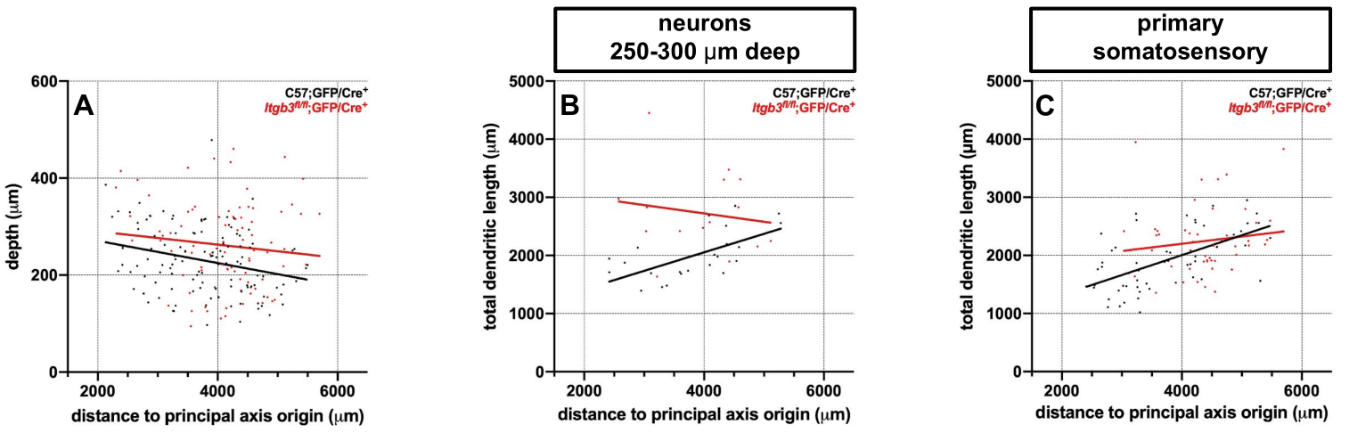


**Additional Figure 5. Somatic depth and region are not important factors when comparing dendritic morphology among targeted layer II/III neurons.** (A) Cortical position along a tangential gradient is weakly correlated with somatic depth in C57;GFP/Cre^+^ neurons, and is not correlated among *Itgb3^fl/fl^*;GFP/Cre^+^ neurons (C57;GFP/Cre^+^ R^2^ = 0.074, P = 0.003, N = 116 neurons; *Itgb3^fl/fl^*;GFP/Cre^+^ R^2^ = 0.009, p = 0.361, N = 86 neurons). (B) When somatic depth is controlled, total dendritic length is still significantly correlated to cortical position along a tangential gradient among C57;GFP/Cre^+^ but not *Itgb3^fl/fl^*;GFP/Cre^+^ layer II/III neurons (C57;GFP/Cre^+^ R^2^ = 0.392, P = 0.0014, N = 23 neurons; *Itgb3^fl/fl^*;GFP/Cre^+^ R^2^ = 0.008, P = 0.747, N = 16 neurons). (C) Within the primary somatosensory cortex, dendritic complexity is correlated with the tangential cortical positions of C57;GFP/Cre^+^ (but not *Itgb3^fl/fl^*;GFP/Cre^+^) layer II/III neurons (C57;GFP/Cre^+^ R^2^ = 0.307, p < 0.0001, N = 53 neurons; *Itgb3^fl/fl^*;GFP/Cre^+^ R^2^ = 0.044, p = 0.152, N = 48 neurons). C57;GFP/Cre^+^ neurons were previously reported in Holley et al. (2018) and re-analyzed for this study.

### Additional Figure 6


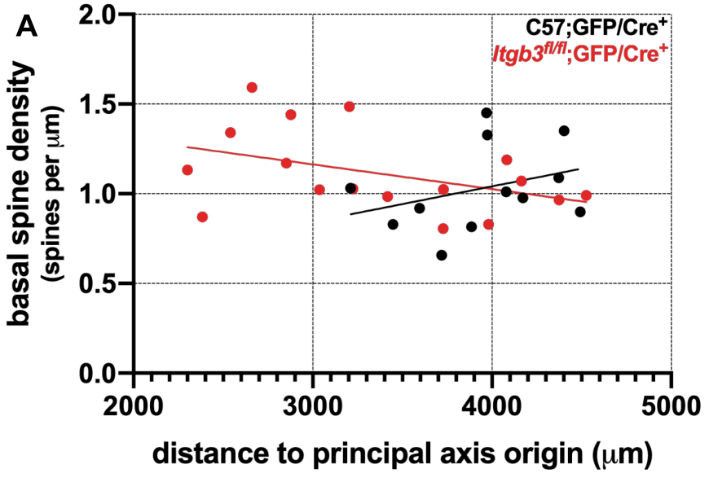


**Additional Figure 6.** In contrast to rostrocaudal position (Figure 4), no correlation exists between total basal dendritic spine density and distance to principal axis origin (*m* = 1.56, *y_0_* = −350 μm) in either C57;GFP/Cre^+^ neurons (*r^2^* = 0.11, p = 0.30) or *Itgb3^fl/fl^*;GFP/Cre^+^ neurons (r*2* = 0.18, p = 0.09).

### Additional Table 1


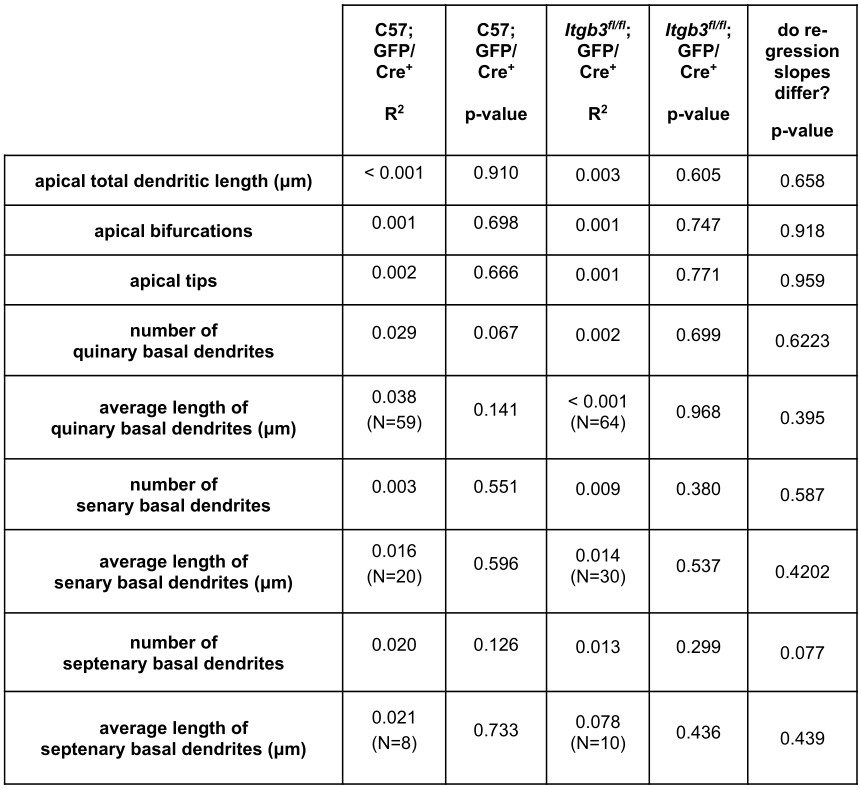


**Additional Table 1. Apical morphology and high-order basal dendritic morphology of neurons are not correlated to their cortical position along a tangential gradient (distance to principal axis origin).** C57;GFP/Cre^+^ neurons were previously reported in Holley et al. (2018) and re-analyzed for this study. C57;GFP/Cre^+^ N = 116 neurons; *Itgb3^fl/fl^*;GFP/Cre^+^ N = 86 neurons.

## Methods for Additional Figure 1

### Mice

To confirm that cell- and tissue-specific excision leads to decreased integrin β3 expression (Morgan *et al*., 2010; Faralli *et al*., 2019), we crossed *Itgb3^fl/fl^* mice with B6.129S2-*Emx1^tm1(cre)Krj^*/J mice (“*Emx1*-Cre”, Jackson Labs #005628, Gorski et al., 2002) to produce a homozygous *Itgb3^fl/fl^*; *Emx1*-Cre^+/+^ line (“cKO”), targeting the conditional knockout of *Itgb3* in excitatory neurons and glia of cortex and hippocampus.

### Golgi-Cox stain

A control experiment was performed to determine whether the floxed *Itgb3* allele alone alters the dendritic morphology of layer II/III pyramidal neurons. Coronal sections of C57 and *Itgb3^fl/fl^* were stained by the Golgi-Cox method using the FD Neurotech Rapid GolgiStain Kit (#PK401). Following a lethal intraperitoneal injection of ketamine (240 mg/kg)-xylazine (48 mg/kg)-acepromazine (1.85 mg/kg), C57 and *Itgb3^fl/fl^* mice at P23 were transcardially perfused with ice-cold 1x PBS followed by 4% PFA. Each brain was immediately dissected and divided into two hemispheres. The hemispheres were then postfixed in 4% PFA for 15 minutes and rinsed with water. The brain was placed in the Rapid GolgiStain Kit impregnation solution (equal parts solution A/B) for 9-11 days. Following incubation in the impregnation solution, the tissue was then placed in solution C and stored for 48 hours. The brain was mounted in agar and sliced into 200 μm sections on a vibrating microtome. Sections were placed in solution D/E (1 part solution D, 1 part solution E, 2 parts Milli-Q water) and allowed to develop for 8 minutes. Sections were then rinsed in water, mounted onto gelatin-coated slides with glycerol, and sealed with nail polish. Layer II/III neurons were identified by their pyramidal dendritic morphology and imaged at 20×/0.75NA with a 1.5× insert on a Nikon Eclipse Ti-2 microscope and at 60×/1.40NA on a Nikon Eclipse TE2000-E confocal microscope. Images were manually analyzed blind to genotype to determine the number of primary basal dendrites on each neuron.

### Western Blot

Following a lethal intraperitoneal injection of ketamine (240 mg/kg)-xylazine (48 mg/kg)-acepromazine (1.85 mg/kg), P23 C57 and cKO mice were transcardially perfused with ice-cold 1x PBS. The cerebral cortex of each mouse was then immediately dissected and flash frozen. Thawed tissue was then lysed with 18 mL 1x Cell Lysis Buffer (Cell Signaling Technology) containing PMSF (Cell Signaling Technologies, 1:200) and Protease Inhibitor (Cell Signaling Technology, 1:100) per gram of tissue. The tissue was then homogenized with a dounce homogenizer and sonicated with a probe sonicator for 30 s. The lysate was centrifuged for 20 min at 13,000 x g. The protein concentration of the supernatant was calculated using a BCA protein assay kit (Cell Signaling Technology). Loading buffer (Cell Signaling Technology) was added to 46 µg of protein and brought up to a volume of 25 µL with water and loaded onto a 10% Tris-glycine wedge mini-gel (Invitrogen) at 250 V for 45 min. The protein was then electro-transferred to a nitrocellulose membrane (Cell Signaling Technology) at 25 V for 1.5 h. The membrane was stained with Ponceau S solution (Sigma-Aldrich) for 5 minutes to check for transfer quality, then destained in Milli-Q water and placed in blocking solution (1x TBST with 5% w/v nonfat dry milk) for 1 h at room temp. The membrane was then transferred to the primary antibody solution (blocking solution containing rabbit anti-β-actin monoclonal antibody, Cell Signaling Technology #13E5, 1:1000, and rabbit anti-Itgb3 (D7X3P) monoclonal antibody, Cell Signaling Technology #13166, 1:1000, overnight at 4 °C. Following incubation with the primary antibodies, the membrane was washed three times with 1x TBST and incubated in secondary antibody solution (blocking solution containing anti-rabbit IgG HRP-linked antibody, Cell Signaling Technology #7074, 1:2000) at room temperature for 1 hour. The membrane was soaked in 1x Signal Fire ECL reagent (Cell Signaling Technology) for one 1 minute prior to imaging.
